# Implication of Surface Properties, Bacterial Motility, and Hydrodynamic Conditions on Bacterial Surface Sensing and Their Initial Adhesion

**DOI:** 10.3389/fbioe.2021.643722

**Published:** 2021-02-12

**Authors:** Sherry Zheng, Marwa Bawazir, Atul Dhall, Hye-Eun Kim, Le He, Joseph Heo, Geelsu Hwang

**Affiliations:** ^1^Department of Preventive & Restorative Sciences, School of Dental Medicine, University of Pennsylvania, Philadelphia, PA, United States; ^2^Center for Innovation & Precision Dentistry, School of Dental Medicine, School of Engineering and Applied Sciences, University of Pennsylvania, Philadelphia, PA, United States

**Keywords:** bacterial adhesion, biofilm formation, material properties, bacterial motility, hydrodynamics, antibiofilm surfaces, bacterial surface sensing

## Abstract

Biofilms are structured microbial communities attached to surfaces, which play a significant role in the persistence of biofoulings in both medical and industrial settings. Bacteria in biofilms are mostly embedded in a complex matrix comprised of extracellular polymeric substances that provide mechanical stability and protection against environmental adversities. Once the biofilm is matured, it becomes extremely difficult to kill bacteria or mechanically remove biofilms from solid surfaces. Therefore, interrupting the bacterial surface sensing mechanism and subsequent initial binding process of bacteria to surfaces is essential to effectively prevent biofilm-associated problems. Noting that the process of bacterial adhesion is influenced by many factors, including material surface properties, this review summarizes recent works dedicated to understanding the influences of surface charge, surface wettability, roughness, topography, stiffness, and combination of properties on bacterial adhesion. This review also highlights other factors that are often neglected in bacterial adhesion studies such as bacterial motility and the effect of hydrodynamic flow. Lastly, the present review features recent innovations in nanotechnology-based antifouling systems to engineer new concepts of antibiofilm surfaces.

## Introduction

Biofilm is a three-dimensional structure formed as a result of microorganism’s surface sensing, initial adhesion to surfaces, followed by subsequent colonization and production of an extracellular polysaccharides matrix (EPS) (Flemming et al., 2016). The development of the biofilm is a sequential process that starts with a loose association of the microorganisms to a surface then converted to strong adhesion. At the final stage of adhesion, the bacterial cell wall is deformed, which reinforces the bacteria’s adhesion toward the surface by positioning the cytoplasmic bacterial molecules closer to the surface. This enables bacteria to interact with surfaces using their bacterial surface molecules in the form of Lifshitz-van der Waals attractive forces (Carniello et al., 2018). Once the microorganisms adhere to a surface, they often aggregate and form microcolonies that maturate over time (An and Friedman, 1998). The structured channels within the biofilm facilitate the exchanges of nutrients and byproducts between the embedded microorganisms and the external environment, which attributes to the microorganisms’ colonization growth and maturation (Flemming et al., 2016). After biofilm maturation, the microorganisms shed and move from the matured biofilm to join another biofilm community or to become a pioneer of a new one (Hall-Stoodley et al., 2004).

A significant feature of the biofilm is that it can form either on a biotic or abiotic surface. Thus, it is deeply associated with a diverse spectrum of industrial biofouling as well as human health problems, such as dental caries, infective endocarditis, cystic fibrosis pneumonia, and peritoneal dialysis catheters infection (Hall-Stoodley et al., 2004). Particularly, 60–70% of all healthcare-associated infections are attributed to biofilm infections in implantable medical devices (Bryers, 2008), thereby it is imperative to develop novel anti-infectious biomaterials. Biofilm formation is essential for microorganisms’ survival since it benefits bacteria; it stimulates bacterial growth and acts as a barrier that protects the embedded microorganisms from environmental challenges and administered antimicrobials (Lebeaux et al., 2014). During the biofilm maturation, the EPS matrix enhances cell adhesion and cohesion that promote both microbial accumulation onto a surface and the development of densely packed cell aggregates, resulting in a highly structured and adherent biofilm. As such, once biofilms are established, it becomes extremely difficult to kill embedded bacteria or mechanically remove biofilms from surfaces. Therefore, interrupting the bacterial surface sensing mechanism and their initial binding process to surfaces is essential to effectively prevent biofilm-related problems.

**FIGURE 1:**
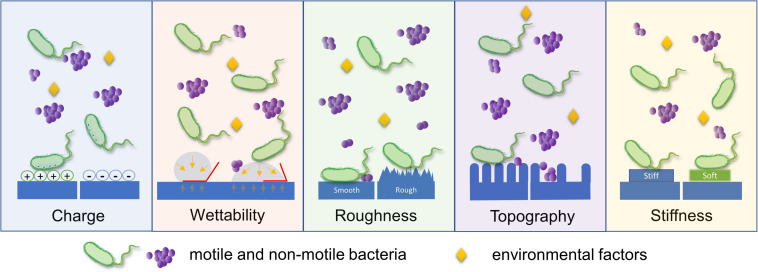
Schematic illustration of various surface parameters that influence bacterial adhesion. Bacterial adhesion is governed by diverse surface properties, including surface charge density, wettability, roughness, topography, and stiffness. This diagram describes the major aspect of bacterial response to a single surface parameter.

Highlighting that biofilm formation is initiated by bacterial adhesion to a surface, bacterial sensing and responding to surfaces have been widely studied. There are many factors affecting the process of bacterial adhesion to a surface; duration of exposure of bacteria to surfaces, population of inoculated bacteria, bacterial characteristics (e.g., cell wall components, appendages, and motility), and type/richness of nutrients could affect. Surface properties of the substrate, such as surface charge density, wettability, roughness, stiffness, and surface topography are also considered important factors governing initial bacterial adhesion to surfaces (as illustrated in Figure 1) (An and Friedman, 1998; Song et al., 2015; Carniello et al., 2018; Li et al., 2019; Chien et al., 2020). From that aspect, there were many attempts to explain the mechanism of bacterial binding to surfaces using physicochemical approaches such as thermodynamic theory, Lifshitz-van der Waals, and electrostatic-double layer interactions (Carniello et al., 2018). On the other hand, various strategies have been developed to prevent biofilm formation at an early stage, either by engineering anti-adhesive surface properties or introducing antibacterial elements to a surface (Zhang et al., 2018; Chi et al., 2019; Li et al., 2019).

In this review, we aim to summarize and discuss the recent publications regarding bacterial sensing and responding to individual/combined surface properties. Furthermore, we introduce other important but not yet sufficiently highlighted parameters in regards to bacterial interaction with surfaces, such as bacterial motility and the effect of hydrodynamic flow. Lastly, we give a quick glance at the nano-science and new anti-adhesion approaches.

## Surface Properties

### Surface Charge Density

Surface charge density is one of the important surface properties that can determine bacterial adhesion onto surfaces. According to Renner and Weibel (2011), van der Waals force and electrostatic interactions are one of the major forces in bacterial adhesion onto material surfaces. Many have proposed the mechanism by which surface charge affects bacterial initial adhesion. Considering that bacteria usually possess a net negative charge due to carboxyl, amino, and phosphate groups on their cell wall surfaces (Rijnaarts et al., 1999), more adhesion on positively charged surfaces were often observed (Gottenbos et al., 1999; Zhu et al., 2015; Kovacevic et al., 2016; Guo et al., 2018; Oh et al., 2018; Chen et al., 2019). An early study on the effect of surface charge on bacterial adhesion showed that initial adhesion and growth of *Pseudomonas aeruginosa* on positively charged poly(methacrylates) were facilitated 2-fold compared to negatively charged surfaces (Gottenbos et al., 1999). Since then, similar observations have been reported by testing different bacteria and surfaces. For example, Zhu et al. (2015) demonstrated greater adhesion of *Pseudomonas, Escherichia coli, and Staphylococcus aureus* on positively charged poly(acrylic acid) (PAA) and poly(diallyldimethylammonium chloride) (PDADMAC) surfaces. Increased bacterial adhesion on positively charged surfaces was also observed for *P. aeruginosa* on a different type of positively-charged poly(allylamine hydrochloride) (PAH) multilayers compared to negatively charged poly(styrenesulfonate) (PSS) multilayers (Kovacevic et al., 2016). The binding trend of *S. aureus* and *E. coli* onto polyelectrolyte multilayers (PEM) was also primarily controlled by surface charge, showing reduced bacterial adhesion on the negatively charged surface (Guo et al., 2018).

Interestingly, some of the studies showed that surface charge density not only affects the initial bacterial attachment but also the subsequent biofilm accumulation on material surfaces (Ueshima et al., 2002; van Merode et al., 2006; Kao et al., 2017; Shen et al., 2020). It’s worth noting, however, that the trends for initial attachment and later biofilm formation were not always coincident. For example, *E. coli* adhered more to positively charged surfaces, but the high charge density induced lower cell viability and retarded biofilm growth later on (Terada et al., 2012). The structure and strength of biofilms on differently charged surfaces were also varied; *E. coli* biofilm on negatively charged surfaces was heterogeneous, sparse, mushroom-shaped, and less shear resistant, while that on positively charged surfaces was homogenous, dense, uniform, and greater shear resistant (Terada et al., 2012).

While numerous studies demonstrated that negatively charged surfaces reduce bacterial adhesion to its surface, the uncertainty on the trend between charge and bacterial attachment is further complicated by research that shows mixed results. It has been reported that some bacteria have an ability to overcome electrostatic repulsion despite net negative charge on their surface and they even bind tightly to negatively charged surfaces due to their surface appendages, such as fimbriae (Ueshima et al., 2002). Also, lipopolysaccharide (LPS), a bacterial surface polymer of Gram-negative bacteria (e.g., *P. aeruginosa* and *E. coli*), was able to assist their adhesion to negatively charged surfaces (Rzhepishevska et al., 2013). Oppositely, reduced adhesion of *Streptococcus mutans* to a positively charged surface has been reported (He et al., 2019). Frueh et al. (2014) found that the negative ends of PEM films reduced the attachment of Gram-positive bacteria, and the positive ends reduced the attachment of Gram-negative bacteria. On the other hand, both positively and negatively charged polystyrene plates were found to decrease *P. aeruginosa* adhesion compared to unmodified plates (Kao et al., 2017). Therefore, further research is needed to investigate to fully understand how surface charge density affects adhesion and biofilm formation of different types of bacteria. Studies regarding surface charge density and its effects on bacterial adhesion and biofilm formation are summarized in Table 1.

**TABLE 1:** Influence of surface charge on bacterial adhesion and biofilm growth.

Microorganism	Surface material	Influence on adhesion/biofilm growth	References
*E. coli*	Polythylene sheets modified by radiation-induced graft polymerization (RIGP) of an epoxy-group containing monomer glycidyl methacrylate (GMA)	Increased bacterial adhesion on positively charged surface, biofilm structure was dense, homogenous and uniform	Terada et al., 2012
*Pseudomonas, E. coli*, and *S. aureus*	Positively charged poly(acrylic acid) (PAA) and poly(diallyl dimethyl ammonium chloride) (PDADMAC)	Increased bacterial adhesion on a positively charged surface	Zhu et al., 2015
*P. aeruginosa*	Poly(allylamine hydrochloride)/sodium poly(4-styrenesulfonate) (PAH/PSS) polyelectrolyte multilayers	Increased bacterial adhesion on a positively charged surface	Kovacevic et al., 2016
*S. aureus* and *E. coli*	Gold coated plates with thin thiol layers of 1-octanethiol, 1-decanethiol, 1-octadecanethiol, 16-mercaptohexadecanoic acid, and 2-aminoethanethiol hydrochloride	Increased bacterial adhesion and biofilm thickness on hydrophilic substrates with positive surface charge	Oh et al., 2018
*E. coli*	Layer-by-layer (LbL) assembly of cationic polyvinylamine (PVAm)/anionic cellulose nanofibril/PVAm	Increased bacterial adhesion and bacterial viability as surface charge increases	Chen et al., 2019
*S. aureus* and *E. coli*	Polyethylenimine multilayers	Reduced bacterial adhesion on a negatively charged surface	Guo et al., 2018
*S. mutans*	Chimaeric peptide-mediated nanocomplexes of carboxymethyl chitosan/amorphous calcium phosphate (CMC/ACP)	Reduced bacterial adhesion on a positively charged surface	He et al., 2019
*S. aureus, P. aeruginosa* and *E. coli*	Hydroxide coated titanium alloy (Ti-OH) discs coated with (3-aminopropyl) triethoxysilane, resulting in hydrophobic alkyl chain and positively charged amino group	Reduced bacterial adhesion and subsequent growth on a positively charged surface	Shen et al., 2020
*P. aeruginosaa*	Positively charged poly(allylamine hydrochloride) (PAH) and negatively charged poly(styrenesulfonate) (PSS)	Reduced bacterial adhesion to both positively and negatively charged plates	Kao et al., 2017
*Bacteria in fresh water*	Polyelectrolyte multilayers (PEM) on PDMS	Selective reduction of bacterial adhesion to charged surfaces	Frueh et al., 2014

### Surface Wettability

As the point of contact between bacteria and the bulk of a material, the surface plays a pivotal role in promoting or preventing bacterial adhesion. Surface wettability is a central property that governs the interactions between solid and liquid phases in biological systems. Briefly, the liquid phase “wets” the surface of a solid surface by maximizing its area in contact with the surface. This in turn increases the interaction between the liquid and the solid surface. In general, surfaces with low surface energy and liquids with high surface tension tend to reduce surface wettability. On the other hand, surfaces with high surface energy and liquids with low surface tension tend to increase surface wettability (Song et al., 2019; Jothi Prakash and Prasanth, 2021).

**FIGURE 2:**
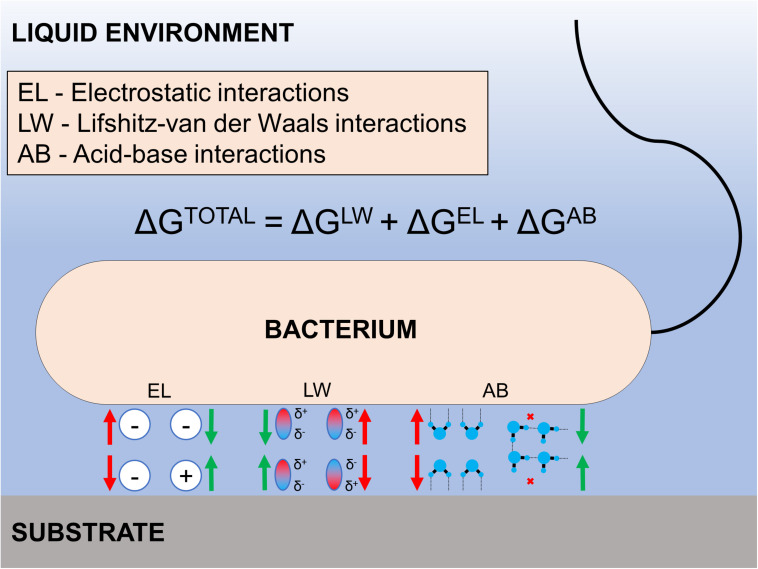
Illustration of the extended DLVO theory. The total interaction energy between the bacterium and a substrate is the sum of the electrostatic double layer interactions, Lifshitz-van der Waals interactions, and acid-base interactions. Each energy component can be either attractive or repulsive depending on the surface properties of the bacterium and substrate in aquatic environmental conditions.

The intrinsic wettability of a surface depends primarily on its surface energy and roughness (Song et al., 2019). Various models have been utilized to explain the relationship between surface energy/interfacial interaction energy and bacterial adhesion. These models stem from Derjaguin-Landau-Verwey-Overbeek (DLVO), extended-DLVO, and thermodynamics-based approaches (Bos et al., 1999). For example, a thermodynamic approach based on the pairwise interplay of surface free energies among surface, fluid, and bacteria was utilized to explain bacterial adhesion phenomena (Rigolin et al., 2019; Bilgili et al., 2020; Ruan et al., 2020). The adhesion energy can be estimated as the sum of the Lifshitz-van der Waals interactions, the electric double layer interactions, and the often-overpowering acid-base interactions. A resultant negative free energy favors bacterial adhesion, while positive free energy may inhibit it. Overall, the key benefit of using a thermodynamic approach is its usefulness in explaining generic like-to-like observations, such as bacteria with hydrophobic cell surfaces favor hydrophobic material surfaces while those with hydrophilic cell surfaces favor hydrophilic material surfaces (Krasowska and Sigler, 2014). Similarly, bacterial adhesion is often suitably described by DLVO and its derivative theories (Hwang et al., 2010, 2012; Carniello et al., 2018; Zhang et al., 2019) since the size of most bacteria (0.5–2 μm) is close to the range of colloidal particles (Hori and Matsumoto, 2010). Briefly, the relative strengths of van der Waals and Coulomb interactions enable bacterial adhesion to surfaces depending on the bacterium-surface distance and ionic strength of the surrounding fluid. In general, higher ionic strengths favor bacterial adhesion (Sheng et al., 2008) while lower ionic strengths present an insurmountable energy barrier that prevents adhesion (Hori and Matsumoto, 2010). Notably, it is critical to consider the polar interaction energy for the aqueous system to fully account for the total interactive energies between bacterium and counter substrate surface (extended DLVO theory; see Figure 2).

However, it is necessary to point out that researchers should be cautious in drawing broad conclusions on the impact of wettability on bacterial adhesion. There are conflicting findings on the initial adhesion of bacteria on surfaces with moderate hydrophobicity and hydrophilicity (Lorenzetti et al., 2015; Mandracci et al., 2015; Wassmann et al., 2017; Yuan et al., 2017; El-Chami et al., 2020; Pajerski et al., 2020; Verhorstert et al., 2020). Nevertheless, engineered materials and surface treatments can result in extreme water contact angles – either superhydrophobic or superhydrophilic surfaces that can limit bacterial adhesion (Moazzam et al., 2016; Qin et al., 2019; Naderizadeh et al., 2020; Ozkan et al., 2020; Montgomerie and Popat, 2021). For example, the adhesion of *S. aureus* on superhydrophobic surfaces (commercial polyurethane sponges modified by zinc oxide and copper nanoparticles) was severely impaired (Ozkan et al., 2020). Similarly, the adhesion of *E. coli* on superhydrophilic surfaces (stainless steel plates coated with TiO_2_) was greatly reduced (Yoon et al., 2014). Overall, low surface energy is the primary means of attaining an anti-wetting surface. However, higher levels of anti-wetting surfaces can only be achieved by the implementation of surface roughness (Song et al., 2019). Studies regarding surface wettability and its effects on bacterial adhesion and biofilm formation are summarized in Table 2.

**TABLE 2:** Influence of surface wettability on bacterial adhesion and biofilm growth.

Microorganism	Surface material	Parameter tested	Influence on adhesion/biofilm growth	References
*F. nucleatum, P. gingivalis*, and *S. sanguinis*	Aged titanium and zirconium oxide discs	Contact angles and surface free energy	Increased surface free energy values led to increased biofilm adhesion on zirconium oxide surfaces as compared to titanium surfaces	Rigolin et al., 2019
*S. mutans* and *S. mitis*	Various commercial bulk-fill composite dental resins	Contact angles and surface free energy	Bacterial adhesion increased with higher surface free energies	Bilgili et al., 2020
*Sphingomonas* sp. GY2B	Montmorillonite	Ionic strength of surrounding liquid, DLVO components	Adhesion was driven by long-range DLVO forces at low ionic strength and short-range van der Waals and hydrophobic interactions at high ionic strength	Ruan et al., 2020
*E. coli*	Titanium substrates with TiO_2_-anatase nanostructured coating	Contact angles	Increased hydrophilicity of treated surfaces reduced bacterial adhesion	Lorenzetti et al., 2015
*S. mutans* and *S. mitis*	Silicon-oxygen thin films on dental composite resins	Contact angles	Hydrophilic coated samples reduced *S. mitis* adhesion. *S. mutans* adhesion was not affected by wettability	Mandracci et al., 2015
*S. epidermidis, S. aureus, P. aeruginosa*, and *E. coli*	Oxygen-plasma treatment of conductive graphene sheets	Contact angles, surface free energy, and DLVO components	Short time of plasma modification led to significant increases in work function, surface free energy, hydrophilicity, and bacterial adhesion	Pajerski et al., 2020
*S. aureus* and *E. coli*	Poly-4-hydroxybutyrate (P4HB) and polypropylene	Contact angles	Hydrophilic substrates had lower adhesion than hydrophobic substrates	Verhorstert et al., 2020
*E. coli*	Polystyrene	Contact angles and surface energy	Moderate hydrophobicity led to the highest bacterial adhesion. Superhydrophilic substrates limited bacterial binding	Yuan et al., 2017
*S. sanguinis* and *S. epidermidis*	Titanium and zirconium oxide dental implants	Contact angles	Hydrophobic surfaces favored adhesion of *S. epidermidis*. *S. sanguinis* adhesion was not affected by wettability	Wassmann et al., 2017
*S. aureus* and *P. aeruginosa*	Bare, polyurethane, and parylene-coated titanium	Contact angles	Higher contact angles for parylene-coated samples in comparison to bare samples led to reduced bacterial adhesion	El-Chami et al., 2020
*E. coli*	Stainless steel with TiO_2_ coating	Contact angles	Superhydrophilic surfaces greatly reduced bacterial adhesion	Yoon et al., 2014
*E. coli*	Polydimethylsiloxane (PDMS) layered with 2-methacryolyl phosphorylcholine (MPC)	Contact angles	Superhydrophilicity of MPC layer significantly lowered bacterial attachment	Qin et al., 2019
*P. aeruginosa*	Polyvinyl chloride	Contact angles	Superhydrophobic substrates delayed initial bacterial adhesion by up to 24 h	Loo et al., 2012
*P. aeruginosa, S. epidermidis*, and *S. aureus*	Silanized aluminum	Contact angles and surface free energy	Superhydrophobic modification significantly reduced bacterial attachment	Moazzam et al., 2016
*E. coli* and *S. aureus*	Titania nanoflowers on titanium alloy	Contact angles	Superhydrophobic surfaces significantly reduced bacterial adhesion	Montgomerie and Popat, 2021
*E. coli, S. aureus*, and *P. aeruginosa*	Aluminum foil substrates coated with polyfurfuryl alcohol, perfluorinated acrylic copolymer and Silica nanoparticles	Contact angles	Superhydrophobic coatings exhibited very low bacterial adhesion	Naderizadeh et al., 2020
*S. aureus*	Polyurethane sponges with zinc oxide and copper nanoparticles	Contact angles	Superhydrophobic characteristics reduced bacterial adhesion over 4 days	Ozkan et al., 2020

### Surface Roughness

The effect of surface roughness on bacterial adhesion and biofilm formation has been extensively investigated. Surface roughness increases the surface area available for bacterial attachment and provides a scaffold for adhesion (Yoda et al., 2014). Additionally, rough surfaces can provide protection for bacteria against shear forces (Bollen et al., 1996), thereby resisting the detachment of bound bacteria. Therefore, the general consensus is that as surface roughness increases, bacterial adhesion and biofilm formation also increases (Xing et al., 2015; Yu et al., 2016; Yao et al., 2020). For instance, the adhesion of *Staphylococcus epidermidis, P. aeruginosa*, and *Ralstonia pickettii* on rougher surfaces with larger surface areas were remarkably higher, compared to the smoother surfaces (James et al., 2019). A similar pattern was also observed from oral bacteria (such as Streptococci) exhibiting that bacterial adhesion was significantly enhanced to rougher surfaces, regardless of surface materials (Yu et al., 2016; Nogueira et al., 2017; Abdalla et al., 2020). Furthermore, an *in vivo* study reported similar results showing that biofilm accumulation increased in proportion to the nanoscale surface roughness of titanium disc samples (ranging from 29 to 214 nm) placed intraorally via a removable splint model (Xing et al., 2015). Furthermore, biofilm accumulation was more pronounced on the irregularly textured titanium disc surfaces (surface roughness of 190 and 214 nm) than the flat and grooved surfaces (<113 nm) (Xing et al., 2015).

Some studies have proposed the idea of a threshold arithmetic average roughness (Ra) of 0.2 μm. For example, an *in vitro* study claimed that threshold values for the adhesion of *S. mutans* and *Streptococcus sobrinus* to composite resin surfaces were estimated between 0.15 and 0.35 μm (Park et al., 2019). However, other studies contain findings that disprove the existence of such a threshold. Bollen et al. (1996) reported that smoothing a surface past the threshold had no significant reductive effect on bacterial adhesion by testing two types of dental implant abutments *in vivo* (Ra = 0.21 μm and Ra = 0.06 μm) using a split-mouth design over 12 months. Other studies showed increased bacterial adhesion in proportional to surface roughness even in the range lower than the proposed threshold (0.2 μm). As observed by Yu et al. (2016), an increase in surface roughness of zirconia discs from fine (Ra = 11.89 ± 1.68 nm) to coarse (Ra = 23.94 ± 2.52 nm) was positively correlated with adhesion strength and the number of attached bacteria. Furthermore, Yoda et al. (2014) reported that the number of adhered *S. epidermidis* on various biomaterials was higher in the course group (Ra = 7.2–30.0 nm) compared with the fine group (Ra = 1.8–8.5 nm). Various studies continue to observe different findings with regards to the threshold roughness of Ra = 0.2 μm. Thus, the existence of a threshold roughness is currently debatable.

Although surface roughness is often positively correlated with the degree of bacterial adhesion and biofilm formation, higher surface roughness in some cases resulted in reduced bacterial adhesion (Wu et al., 2018; Matalon et al., 2020). For example, the adhesion of *P. aeruginosa* and *S. aureus* on unpolished stainless steel samples (Ra = 172.5 nm) were decreased significantly compared with the electropolished smoother surfaces with average roughness ranging from 45.2 to 84.4 nm (Wu et al., 2018). Moreover, the qualitative images of the tested samples revealed the presence of scattered single bacterial cells attached to the rough stainless steel surface, while clusters of the bacterial cells were observed on the smooth, electropolished surfaces (Wu et al., 2018). This finding illustrates that the nano-rough surfaces can restrain the bacterial adhesion by decreasing the contact area between the bacteria and surface (Wu et al., 2018). In contrast, one study reported that surface roughness affected bacterial adhesion selectively; higher surface roughness increased the adhesion of *Streptococcus sanguinis*, while there was no difference in *S. epidermidis* adhesion (Wassmann et al., 2017).

Another study found that surface root-mean-square roughness (Rq) of Titania up to 20 nm was linked with increased bacterial adhesion and biofilm formation (Singh et al., 2011). However, further increasing the roughness to 25 nm resulted in a significant decrease in bacterial adhesion and biofilm formation. This phenomenon was explained as the passivation and flattening effects induced by bovine serum albumin adsorption on the surface of the Titania. Increased protein adsorption to the rougher surface created an intermediary layer between bacteria and Titania, which limited the interaction of bacteria to the nanostructured surface (Singh et al., 2011). Puckett et al. (2010) observed a similar phenomenon when comparing fibronectin adsorption and bacterial adhesion on a nano-rough titanium surface to a smooth, unmodified Ti surface. The nanorough Ti surface exhibited reduced adhesion of *S. aureus, S. epidermidis*, and *P. aeruginosa* than those to smooth Ti surface. The authors explained these findings by the effect of the nanometer surface roughness in enhancing the adherence of fibronectin protein, which has been claimed to decrease bacterial attachment (Puckett et al., 2010). They also found that it was possible to reverse the surface ability to repel bacterial adhesion by altering the chemical composition or crystallinity of the nano- featured Ti surfaces. It indicates that bacterial response to surface roughness can be varied by other factors, such as surface priming by proteins and changes in surface chemistry.

Like other surface properties, there are other variables that can affect the bacterial behavior and adherence toward the surface in response to its roughness, including differences in the bacterial species tested and mediums used in experiments (surface priming proteins). Those need to be accounted for precisely evaluating the effect of surface roughness on bacterial adhesion. Studies regarding surface roughness and its effects on bacterial adhesion and biofilm formation are summarized in Table 3.

**TABLE 3:** Influence of surface roughness on bacterial adhesion and biofilm growth.

Microorganism	Surface material	Surface roughness	Influence on adhesion/biofilm growth	References
*S. epidermidis*	Oxidized zirconium-niobium alloy (Oxinium), cobalt-chromium-molybdenum alloy (Co-Cr-Mo), titanium alloy (Ti-6Al-4 V), commercially pure titanium (Cp-Ti) and stainless steel (SUS316L)	Ra: 1.8–8.5 nm (Fine) Ra: 7.2–30.0 nm (Coarse)	Increased bacterial adhesion in proportion to surface roughness	Yoda et al., 2014
Intraoral biofilm	Titanium Zirconium	Ra: 29–214 nm	Increased biofilm accumulation in proportion to surface roughness	Xing et al., 2015
*S. mutans*	Zirconia	Ra: 11.89 ± 1.68 nm (Fine) Ra: 23.94 ± 2.52 nm (Coarse)	Increased early bacterial adhesion in proportion to surface roughness	Yu et al., 2016
*S. mutans*	Dental enamel (sound enamel, enamel surface treated with laser only, treated with laser and fluoride varnish)	Sa: 2–3 μm (laser and fluoride varnish) <2 μm (sound enamel, and laser only)	Increased bacterial adhesion on roughened enamel surface treated with laser and fluoride varnish	Nogueira et al., 2017
*S. epidermidis, P. aeruginosa*, and *Ralstonia pickettii*	Breast implants materials. [Natrelle^®^ (Smooth), SmoothSilk^®^/SilkSurface^®^ (Silk), VelvetSurface^®^ (Velvet), Siltex^®^, and Biocell^®^]	Not reported.	Increased bacterial adhesion on rougher surfaces	James et al., 2019
*S. mutans*	Ceramics (Feldspathic ceramic, lithium disilicate glass IPS e.max and zirconia reinforced lithium silicate) (Roughened and polished)	Ra: 4.4–4.78 nm (rough) Ra: 1.65–2.07 nm (smooth)	Increased bacterial adhesion on rougher ceramic material	Abdalla et al., 2020
*S. epidermidis*	Titanium (smooth and double acid etched)	Ra: around 4,700 nm (double acid etched titanium)	Increased bacterial adhesion on higher roughness surface with double acid-etched titanium	Kunrath et al., 2020a
*S. epidermidis*	Titanium (Ti) and Zirconia (Zr)	Sa: 0.12 ± 0.01 μm and 0.08 ± 0.00 μm (Machined Ti and Zr) Sa: 2.67 ± 0.20 μm and 0.60 ± 0.05 μm (micro-textured Ti and Zr)	Increased initial bacterial adhesion on rougher microtextured Titanium and Zirconia surfaces.	Kunrath et al., 2020b
*Prevotella intermedia*	Ceramics (leucite-based glass ceramic, lithium disilicate-based glass ceramic, glass ceramic based on zirconia-reinforced lithium silicate, and monolithic zirconia)	Ra: 0.67–0.90 μm (control) Ra: 0.76–1.09 μm (glazed) Ra: 1.04–1.50 μm (grinded surface with bur)	Increased bacterial adhesion on rougher grinded ceramics surface	Poole et al., 2020
*S. mutans*	Enamel surface	Ra: 19.48 ± 3.34 nm (sound enamel) Ra: 150.53 ± 12.54 nm – 341.05 ± 74.14 nm (rougher enamel surface induced by gradual increase in etching time)	Increased adhesion forces for *S. mutans* to rougher enamel surface	Wang et al., 2020
*S. mutans, S. sobrinus*	Dental composite resin	Smooth: 0.15 μm Rough: 1.45 and 0.62 μm	Reduced bacterial adhesion on the smooth surface (roughness values of around 0.15 μm)	Park et al., 2019
*Lactobacillus rhamnosus*	Gold (smooth/nano-rough and micro-rough/sub micro- rough)	Ra: 2.4 nm (smooth/nano-rough) Ra: 7.0 nm (micro-rough/sub micro- rough)	Reduced bacterial adhesion and biofilm density on smoother gold surface.	Grudzień et al., 2020
*Porphyromonas gingivalis*	Titanium (Ti) (control and laser treated)	Ra: 0.167 μm (controlled, untreated Ti) Ra: 0.142 μm (laser treated Ti)	Reduced bacterial adhesion on the smooth surface treated by laser	Yao et al., 2020
*S. sanguinis*	Cr-Co base metal discs, zirconia discs, and lithium disilicate discs	Sa: 0.36 ± 0.12 μm, 0.638 ± 0.24 μm, and 1.23 ± 0.42 μm	Increased bacterial accumulation on the smoothest surface (Cr-Co metal).	Matalon et al., 2020
*P. aeruginosa* and *S. aureus*	Stainless steel	Ra: 172.5 nm (unpolished) Ra: 84.4–45.2 nm (electropolished)	Reduced bacterial adhesion on the rough surface	Wu et al., 2018
*S. sanguinis* and *S. epidermidis*	Zirconia and titanium	Ra: 0.09 ± 0.02 μm and 0.05 ± 0.00 μm (smooth Ti and Zr) Ra: 2.98 ± 0.31 μm and 1.32 ± 0.10 μm (rough Ti and Zr)	Surface roughness did not influence the adhesion of *S. epidermidis*, while higher surface roughness increased the adhesion of *S. sanguinis*	Wassmann et al., 2017

*Ra indicates arithmetical mean height of a line (2D roughness profile) and Sa indicates arithmetical mean height of a surface (3D areal roughness parameter).*

### Surface Topography

Bacteria are capable of sensing mechanical cues associated with surfaces, such as the topography of surfaces (Cheng et al., 2019). In general, microscale features, in the same order as bacteria, impact their attachment through hydrodynamics whereas nanoscale features impact their attachment through chemical gradients, physicochemical forces, and cell membrane deformation (Cheng et al., 2019). Alterations in surface topography are also known to affect the expression of bacterial adhesins (Rizzello et al., 2012, 2013).

Non-covalent interactions between bacteria and substrate surfaces can be directly influenced by the structural surface features (Lutey et al., 2018). Briefly, surfaces with a characteristic feature of a dimension larger than a single bacterium provide greater available contact area and sheltering for the microorganism, thereby resulting in an enhanced bacterial attachment (Scardino et al., 2008; Helbig et al., 2016). This effect is more pronounced on engineered surfaces that have well-defined features and spacing. It is well documented that microbes tend to preferentially align to valley and pillar structures to maximize attachment area (Perera-Costa et al., 2014; Vasudevan et al., 2014). Oppositely, many surfaces with a smaller feature than a single microorganism exhibited reduced bacterial adhesion (Yang et al., 2015; Helbig et al., 2016; Xu and Siedlecki, 2017; Schwibbert et al., 2019; Tang et al., 2021).

**FIGURE 3:**
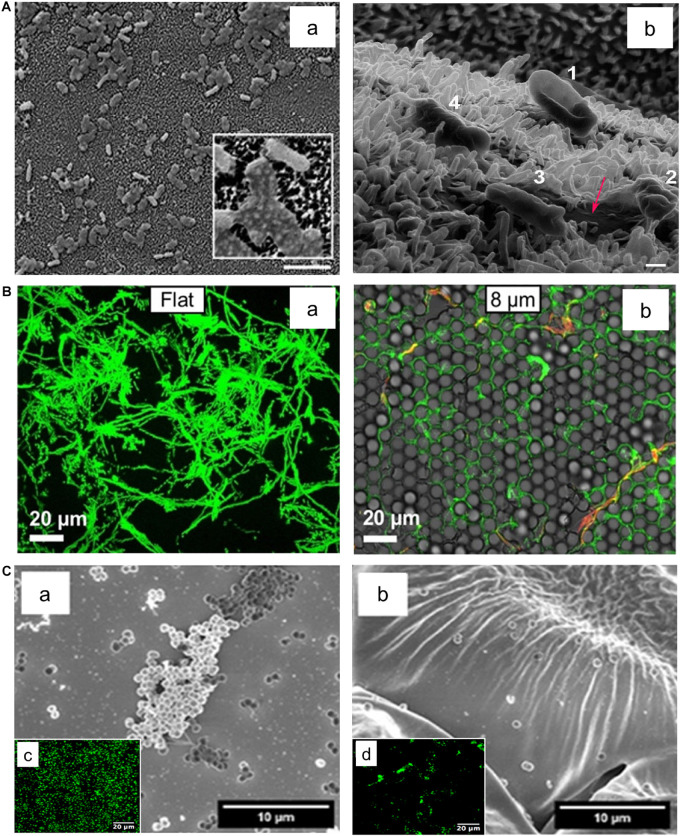
Effect of surface topography on bacterial adhesion. **(A)** *E. coli* bacteria attached on textured surface of dragonfly wing **(a)**. The Helium ion microscopy image **(b)** indicate progressive dying stages of *E. coli* on the dragonfly wing starting with No. 1 where the cell attached to the surface and its membrane deforms, ending with No. 4 where the cell membrane lost its integrity and cell sank into nanopillars. Scale bar is 200 nm. Adapted with permission from Bandara et al. (2017) Bactericidal Effects of Natural Nanotopography of Dragonfly Wing on *Escherichia coli.* ACS Appl Mater Interfaces 9, 6,746–6,760. Copyright © 2017, American Chemical Society. **(B)** Comparison of *P. aeruginosa* adhesion on flat **(a)** and textured surfaces **(b)**. *P. aeruginosa* appears to cover a smaller surface area on the textured surface with features in 8 μm range in comparison to the flat surface. Adapted with permission from Chang et al. (2018) Surface Topography Hinders Bacterial Surface Motility. ACS Appl Mater Interfaces 10, 9,225–9,234. Copyright © 2018, American Chemical Society. **(C)** Adherence of *S. epidermidis* on flat **(a)** and rose petal-textured **(b)** surfaces after 2 h incubation. SEM **(a,b)** and fluorescence microscopy **(c,d)** images of *S. epidermidis* showed a decreased amount of bacterial adhesion on the textured surface. Adapted with permission from Cao et al. (2019) Hierarchical Rose Petal Surfaces Delay the Early-Stage Bacterial Biofilm Growth. Langmuir 35, 14,670–14,680. Copyright © 2019, American Chemical Society.

Recently, several natural surfaces of plants and animals have shed light on the new concept of an antifouling strategy. These include lotuses (Barthlott and Neinhuis, 1997; Bhushan et al., 2009), cicadae (Ivanova et al., 2012), sharks (Chien et al., 2020), termites (Watson et al., 2010), geckos (Watson et al., 2015; Li et al., 2017), butterflies (Fang et al., 2007), and dragonfly wings (Bandara et al., 2017). Such surfaces exhibit contaminant-free surfaces due to their unique superhydrophobic physical surface structures and/or some bactericidal activities. Some prominent recent examples are as follows. Chien et al. (2020) have shown that the topography of sharkskin could significantly alter bacterial attachment and biofilm formation. From their study, the analysis of the height profiles highlights the differences in the topography of the sharkskin. Interestingly, the bacterial attachment was substantially increased on the smooth surface over time, while the protruding surface features inhibited further biofilm development on its surface. Similarly, the micro-/nano-structures on gecko skin exhibited superhydrophobic properties that act as an anti-wetting barrier, resulting in extremely low adhesion (Watson et al., 2015). Gecko skin also revealed antibacterial activity against Gram-negative bacteria, such as *Porphyromonas gingivalis*, and the nanoscale tips on their hair can efficiently kill oral pathogenic bacteria such as *S. mutans* and *P. gingivalis* (Li et al., 2017). Similarly, cicada wings (Ivanova et al., 2012) and dragonfly wings (Bandara et al., 2017) were also known to cause mechanical rupture of bacteria via the nanoscale topography of their wing features (see Figure 3A for the effect of dragonfly wing on the bacteria integrity). Another study proposed an intriguing mechanism that these infection-free natural surfaces may facilitate physical rupture of bacteria by stretching their membrane upon adhesion (Pogodin et al., 2013).

With the aid of various engineering technologies, hierarchical micro- and nanoscale structures with precisely controlled spacing and height of features on surfaces have been used to develop antifouling surfaces via self-cleaning (Patankar, 2004; Cheng and Rodak, 2005; Ma et al., 2011) as well as killing adhered bacteria (Linklater et al., 2017; Watson et al., 2017; Ma et al., 2020). For example, Linklater et al. (2017) used surface geometries in black silicon with linearly increasing heights to show that smaller, densely-packed pillars exhibited the greatest bactericidal effects against both Gram-positive (*S. aureus*) and Gram-negative (*P. aeruginosa*) bacteria. Similarly, cones (Perni and Prokopovich, 2013) and hemispheres (Chang et al., 2018) were engineered onto surfaces to control bacterial adhesion (see Figure 3B for the effect of engineered surface topography on bacterial adhesion). Furthermore, there have been attempts to develop bio-inspired biofilm-resistant surfaces by utilizing natural surfaces as templates for those antibiofilm applications. Cao et al. (2019) engineered a rose petal-textured surface utilizing UV-epoxy, which could disrupt the adhesion of *S. epidermidis* (Figure 3C). Similarly, Ye et al. (2019) patterned cicada wing-like structures on polyether ether ketone (PEEK) substrates and added a top-layer of catkin-like ZnO nano-slices to effectively kill *S. aureus*. Lastly, Nguyen et al. (2018) used wrinkled gold-coated polystyrene surfaces with nano- and microscale topologies to develop air-water interfacial areas that were unavailable for bacterial attachment for *P. aeruginosa* and *S. aureus*. Some studies exhibited distinct anti-adhesion behaviors depending on the type of bacteria (Lutey et al., 2018) or topography structures (Spengler et al., 2019). Studies regarding surface topography and its effects on bacterial adhesion and biofilm formation are summarized in Table 4.

**TABLE 4:** Influence of surface topography on bacterial adhesion and biofilm growth.

Microorganism	Surface material	Scale of surface topography	Influence on adhesion/biofilm growth	References
*E. coli* and *S. aureus*	Honeycomb-patterned silicon wafers	Microscale	1 μm patterns displayed a significant decrease in bacterial adhesion and a subsequent antibacterial effect	Yang et al., 2015
*S. epidermidis* and *E. coli*	Fuctionalized photoresist on silicon wafers	Microscale	Periodicities in the range of the cell size increased bacterial retention. Smaller periodicities reduced retention	Helbig et al., 2016
*S. epidermidis*	Polyethylene glycol-grafted, textured polyurethane urea films	Microscale	Texturing reduced bacterial adhesion. Chemical grafting further reduced bacterial adhesion	Xu and Siedlecki, 2017
*P. aeruginosa*	Norland Optical Adhesive textured on PDMS	Microscale	Texturing reduced bacterial adhesion	Chang et al., 2018
*S. epidermidis*	Bio-inspired rose petal-textured surfaces (made by UV-epoxy)	Microscale	Texturing reduced bacterial adhesion	Cao et al., 2019
*E. coli* and *S. aureus*	Laser-modified polyethylene	Microscale	Adhesion of *E. coli* was reduced while adhesion of *S. aureus* was not affected	Schwibbert et al., 2019
*E. coli* and *S. aureus*	Sharkskin and its Polymethyl methacrylate (PMMA) replicates	Microscale	Protruding surface features inhibited biofilm development	Chien et al., 2020
*S. epidermidis, S. aureus*, and *P. aeruginosa*	Textured fluorinated alkoxyphosphazene surface	Microscale	Patterning led to significant reductions in bacterial adhesion and biofilm formation	Tang et al., 2021
*P. gingivalis*	Gecko skin	Micro/nano scale	Micro/nanostructures displayed an antibacterial effect	Watson et al., 2015
*S. mutans* and *P. gingivalis*	Gecko skin and equivalent acrylic replicates	Micro/nano scale	Micro/nanostructures disrupted normal bacterial adhesion and prevented biofilms by killing bacteria	Li et al., 2017
*E. coli* and *S. aureus*	Stainless steel with laser-induced surface structures	Micro/nano scale	*E. coli* retention was highest when the characteristic dimensions were much larger than the cell size. *S. aureus* retention was inhibited under the same conditions	Lutey et al., 2018
*P. aeruginosa* and *S. aureus*	Gold-coated polystyrene	Micro/nano scale	Topography led to areas unavailable for bacterial attachment	Nguyen et al., 2018
*P. aeruginosa* and *S. aureus*	NiTi sheets with laser-ablation and fluorination	Micro/nano scale	Biofilm formation was suppressed along with the substantial killing of colonized bacteria	Ma et al., 2020
*E. coli*	Dragonfly wing	Nanoscale	Nanostructures displayed an antibacterial effect	Bandara et al., 2017
*P. aeruginosa* and *S. aureus*	Black silicon	Nanoscale	Smaller and densely packed pillars exhibited bactericidal activity and a subsequent decrease in attached cells	Linklater et al., 2017
*S. aureus*	Etched hydrophobized silicon wafers	Nanoscale	Larger nanostructures led to reduced adhesion. Taller nanostructures did not affect adhesion but had a bactericidal effect	Spengler et al., 2019
*S. aureus*	Cicada wing pattern on polyether ether ketone (PEEK) with zinc oxide coating	Nanoscale	Patterned surfaces led to lower bacterial adhesion, wider antimicrobial range, and longer antibacterial durability	Ye et al., 2019
*E. coli* and *S. aureus*	Laser-nanostructured zirconium-based bulk metallic glasses	Nanoscale	Nanostructuring led to a significant reduction in bacterial adhesion	Du et al., 2020

Despite the ability to engineer surfaces that passively exhibit antiadhesion properties, it is vital to realize that many strategies for biofilm prevention that are based on surface treatments and microstructure may affect initial bacterial attachment transiently. However, there are a few promising studies that may lead to a broad range of antibiofilm clinical and industrial applications using topographical features. For example, Epstein et al. (2012) were able to prevent *P. aeruginosa*, *S. aureus*, and *E. coli* adhesion over 7 days on slippery liquid-infused porous surfaces. It is worth noting that elucidating the effect of surface topography on bacterial attachment often leads to contradictory conclusions as many studies use roughness as the primary descriptor of surface topography while neglecting compounding factors such as surface chemistry (Cheng et al., 2019). Furthermore, cell stiffness seems to play a major role in promoting or limiting a cell’s ability to adapt to the pattern on a surface (Whitehead and Verran, 2006; Lazzini et al., 2017). Hence, it is important to design studies that measure surface properties from several different aspects to validate existing hypotheses.

### Surface Stiffness

Stiffness is another important factor that affects bacterial adhesion and biofilm formation. Young’s modulus, which is defined by the ratio of stress to strain, is a common parameter used to represent stiffness. A low Young’s modulus indicates that the material is softer and more elastic. Previous studies have attempted to test the relationship between bacterial adhesion and surface stiffness. Using poly(ethylene glycol) dimethacrylate (PEGDMA) and agar hydrogels as substrate surfaces and *E. coli* and *S. aureus* as model bacteria, Kolewe et al. (2015) found that bacterial adhesion increases with increasing material stiffness regardless of hydrogel chemistry or adhesion mechanism. In fact, studies that use hydrogels, either poly-(ethylene glycol) hydrogels (Kolewe et al., 2015) or agarose hydrogels (Guegan et al., 2014), all demonstrated a positive correlation between adhesion and stiffness. However, studies using polydimethylsiloxane (PDMS) mostly showed a negative correlation between stiffness and adhesion (Song and Ren, 2014; Song F. et al., 2017; Song et al., 2018; Straub et al., 2019), while one study showed an opposite trend (Peng et al., 2019). In Song and Ren’s study, they tested PDMS surfaces that had a range of Young’s modulus between 0.1 and 2.6 MPa, and found that in addition to bacterial adhesion, bacterial cell size is also negatively correlated with stiffness regardless of surface chemistry, roughness, and electrostatic force (Song and Ren, 2014). Interesting results reported by Siddiqui et al. (2019) showed that the retention rate and adhesion strength were higher on softer surfaces when exposed to external shear stress although similar initial bacterial adhesion rates on PDMS were observed. This suggests stiffness-specific interactions between bacteria and PDMS that require further exploration.

Some studies compared the effect of surface stiffness on Gram-positive and -negative bacteria, which exhibited contrasting conclusions. A positive correlation between adhesion and stiffness was observed for Gram-positive *S. epidermidis* and Gram-negative *E. coli* on PEM films prepared from PAH and PAA (Lichter et al., 2008). On the other hand, a negative correlation is observed for Gram-negative *E. coli* on the stiffer photo-cross-linked PEM made from poly(L-lysine) (PLL), a hyaluronan derivative modified with photoreactive vinylbenzyl groups, and softer non-cross-linked PEM films. In contrast, no correlation was observed for Gram-positive *Lactococcus lactis* on the same material (Saha et al., 2013). Such contrast is likely due to the different range of elastic modulus selected for the material. Lichter’s group chose a range of 1 to 100 MPa, while Saha’s group tested using the surface with an elastic modulus of 30 to 150 kPa. However, both studies attributed the mechanoselectivity of bacteria to their surface appendages like flagella and fimbriae (pili), which are lacking from the non-stiffness-responsive *L. Lactis.* In addition, Saha et al. (2013) also proposed that the thick and rigid peptidoglycan structure surrounding Gram-positive *L. lactis* may reduce its mechanoselectivity compared to Gram-negative *E. coli* that has a thinner and softer peptidoglycan layer.

While many studies have been reported, investigations on the underlying mechanism in this topic are not yet sufficient. Song F. et al. (2017) and Song et al. (2018) determined expressed genes that are involved in the bacterial response to material stiffness during adhesion and biofilm formation of *E. coli* and *P. aeruginosa.* Furthermore, they found that high levels of intracellular cyclic demerit guanosine monophosphate (*c-di-GMP*), a key regulator of biofilm formation, is expressed when *P. aeruginosa* is bound to softer surfaces (Song et al., 2018). Peng et al. (2019) also observed increased levels of *c-di-GMP* that reduce bacteria motility and enhance biofilm development when *E. coli* and *Pseudomonas* sp. adhered on the PDMS surface with higher stiffness. Another study showed that surface priming of collagen and fibronectin weakens bacterial adhesion significantly on both soft and hard PDMS (Siddiqui et al., 2019). Peng et al. (2019) had taken a different approach to understanding whether bacterial adhesion is affected by the inherent surface chemical properties of PDMS or affected by the material stiffness. PDMS surfaces of varying stiffness were coated with cross-linked PDMS-like polymer film to establish the same surface chemistry. They observed similar adhesion counts across the varying stiffnesses for the coated surfaces. In contrast, the uncoated surfaces showed increased adhesion of *E. coli*, its fimbriae mutants, *P. aeruginosa*, and *S. epidermidis*. These results highlighted how the presence of free polymer chains of uncoated PDMS rather than the material stiffness promotes bacterial adhesion. This was the first study suggesting that the bacterial adhesion to viscoelastic surfaces could be attributed to the available PDMS polymer chain ends by interfacial adhesion (Pan et al., 2020). Studies regarding surface stiffness and its effects on bacterial adhesion and biofilm formation are summarized in Table 5.

**TABLE 5:** Influence of surface stiffness on bacterial adhesion.

Microorganism	Surface material	Young’s Modulus	Influence on adhesion/biofilm growth	References
*Bacillus* and *Pseudoalteromonas*	Agarose hydrogels	6.6 and 110 kPa	Reduced bacterial adhesion on softer hydrogels	Guegan et al., 2014
*E. coli* and *S. aureus*	Poly(ethylene glycol) dimethacrylate (PEGDMA) and agar hydrogels	Soft: 44.05 – 308.5 kPa. Intermediate: 1495 – 2877 kPa. Stiff: 5152 – 6489 kPa.	Reduced bacterial adhesion on softer hydrogels	Kolewe et al., 2015
*S. aureus*	poly-*N*-isopropylmethacrylamide based microgel coatings	Soft: 21 ± 8 kPa. Intermediate: 117 ± 20 kPa. Stiff: 346 ± 125 kPa.	Reduced bacterial adhesion on softer microgel with lower cross-linking density	Keskin et al., 2019
*E. coli and Pseudomonas* sp.	PDMS	3.4–278.1 MPa	Reduced bacterial adhesion and levels of c-di-GMP when bound to the softer PDMS	Peng et al., 2019
*E. coli* and *L. lactis*	Photo-cross-linked polyelectrolyte films	30–150 kPa	Increased *E. coli* adhesion on softer films, no effect for *L. lactis* adhesion	Saha et al., 2013
*S. aureus*	Polyacrylamide (PAAm) and poly(ethylene glycol) dimethacrylate (PEGDMA)	0.017–0.654 kPa	Increased bacterial adhesion on softer hydrogels	Wang et al., 2016
*E. coli*	PDMS	0.1–2.6 MPa	Increased bacterial adhesion on softer PDMS	Song F. et al., 2017
*E. coli* and *S. aureus*	Poly(ethylene glycol) (PEG) hydrogels	Soft: 20 kPa. Intermediate: 300 kPa, Stiff: 1,000 kPa.	Increased bacterial adhesion and biofilm formation on softer surface	Kolewe et al., 2018
*P. aeruginosa*	PDMS	0.1–2.6 MPa	Increased bacterial adhesion and levels of c-di-GMP when bound to the softer PDMS	Song et al., 2018
*P. aeruginosa E. coli*	PDMS	4.52 ± 0.09–0.06 ± 0.001 MPa	Increased bacterial adhesion on softer PDMS	Straub et al., 2019
*E. coli, P. aeruginosa*, and *S. epidermidis*	PDMS	64.2–2326.8 kPa	Increased bacterial adhesion on softer PDMS	Pan et al., 2020
*E. coli*	PDMS	0.26 ± 0.01 and 124 ± 36 kPa	Similar initial levels of bacterial adhesion on soft and stiff surfaces but increased bacterial adhesion strength on softer surface	Siddiqui et al., 2019

As shown, most previous studies did not account for other surface properties (e.g., physicochemical properties) of the tested substrates when attempting to examine the role of material stiffness on bacterial adhesion. However, the density of polymer cross-linking, material hydrophobicity, material viscosity and topography of surface with the same stiffness can also be responsible for the differences in bacterial adhesion. Thus, further research regarding the underlying mechanism of bacterial sensing on the materials’ stiffness while considering intrinsic material properties are required.

### Complex Surface Properties

Examining combinations of surface properties may give more significant insights into the mechanics of bacterial adhesion and biofilm formation as surface parameters are intrinsically interdependent. While the dominance of one factor over others is certainly possible, particularly for specifically engineered surfaces, it is often necessary to evaluate several surface parameters simultaneously to validate existing hypotheses.

**FIGURE 4:**
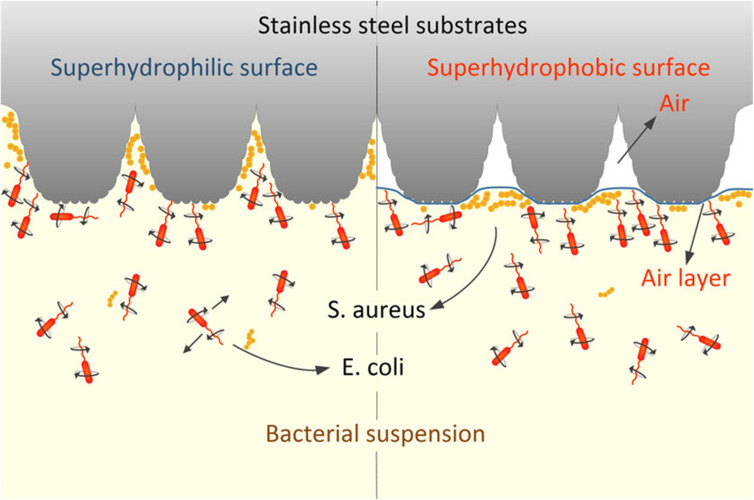
Schematic illustration of bacterial adhesion in response to the superhydrophobic surface. The air entrapment phenomena happened in the presence of increased water contact angle (hydrophobicity), and roughness of the substratum. Reprinted with permission from Pan et al. (2019) Picosecond Laser-Textured Stainless Steel Superhydrophobic Surface with an Antibacterial Adhesion Property. Langmuir 35, 11,414–11,421. Copyright © 2019, American Chemical Society.

Surface wettability and roughness/topography are the representative surface properties that correlate with each other. Generally, when the roughness of a surface increases on a hydrophobic surface, small air pockets may become entrapped within the pores and grooves of the surface (as illustrated in Figure 4). Liquids resting on this interface are not able to penetrate the grooves of the surface and are easily removed. This phenomenon, known as the Cassie-Baxter state, is the basis for superhydrophobic surfaces driven by combined surface roughness and wettability (Giacomello et al., 2012). For example, by applying femtosecond laser ablation to generate similar hierarchical structural topography on titanium surfaces, superhydrophobic surfaces (Contact angle; CA: 166 ± 4°) were created to mimic the features of the lotus leaf *Nelumbo nucifera* (Fadeeva et al., 2011). On initial contact with water, about 50% of the superhydrophobic structured titanium surface area was covered with air pockets trapped in the micro- and nanostructures on the surface. In turn, *P. aeruginosa* was unable to colonize on the superhydrophobic surface. In contrast, *S. aureus* successfully colonized on both the polished, hydrophilic surface (CA: 73 ± 3°) and the superhydrophobic surface with more success (Fadeeva et al., 2011). These contradicting results between species in terms of their ability to colonize on superhydrophobic surfaces might be attributed to the physical properties of the bacterial species; *S. aureus* is a spherical shape, which may require a smaller surface area to bind compared to a rod-shaped bacterium such as *P. aeruginosa*. This indicates that certain species may overcome the antiadhesive properties of superhydrophobic surfaces stemming from a rough surface. In another study, polyvinyl chloride (PVC) was treated with varying concentrations of ethanol and methanol to create surfaces with increasing roughness and hydrophobicity (Loo et al., 2012). The rougher modified PVC surfaces containing micro- and nanoscale structures with measured roughness of 1,250 nm enhanced the hydrophobicity of modified PVCs. The colonization of *P. aeruginosa* was retarded on the superhydrophobic surfaces (150 ± 3°), while colonization was promoted on the untreated, smooth PVC surface (80 ± 1°).

As discussed earlier, surface stiffness and topography are the factors interdependently affecting bacterial adhesion. Interestingly, Arias et al. (2020) found that *E. coli* tend to adhere on PDMS surfaces with variable stiffness in a different pattern when surface topography was introduced to the surface. The wrinkled topography on PDMS surfaces significantly reduced both *E. coli* adhesion and the overall biofilm biomass, regardless of material stiffness. This result suggests that topographical patterns of surfaces may render surface stiffness negligible regarding bacterial adhesion and biofilm accumulation (Arias et al., 2020). Another example of the interdependence of surface parameters that affect bacterial adhesion can be found in a recent study by Yuan et al. (2017). They demonstrated the preferential binding of *E. coli* to moderately hydrophobic polystyrene surfaces by accounting for surface energy, surface roughness, surface charge, and entrapped air. In particular, superhydrophilic substrates with large negative surface charge limited bacterial binding. Additionally, the solid area fraction for superhydrophobic surfaces was reduced due to the entrapped air, which in turn lowered bacterial adhesion. This increased their self-cleaning potential and made the weakly attached bacteria susceptible to removal by washing.

In summary, it is important to comprehensively consider several surface parameters to enhance the antifouling activity of surfaces. Often these multiple parameters are not considered comprehensively, thereby resulting in contradicted bacterial binding results. Thus, the effect of merging multiple surface parameters on bacterial-surface interaction requires further investigation.

## Environmental Factors

### Fluid Dynamics

Hydrodynamic conditions can interfere or enhance bacterial sensing on various surface properties, thereby affecting biofilm architecture, composition, and mechanical strength. Indeed, many infectious biofilms in the human body are formed under dynamic conditions. In the oral cavity, for example, dental plaques are subject to salivary and gingival crevicular fluid flow (Faran Ali and Tanwir, 2012). Furthermore, fluid flow is pertinent in catheter microenvironments, thereby affecting biofilm formation (Weaver et al., 2012; Alves et al., 2020). While hydrodynamic conditions can significantly affect bacterial behavior on surfaces and subsequent biofilm formation, the influence of dynamic conditions on bacterial adhesion and biofilm formation has been often neglected.

According to previous literature, biofilm growth can be facilitated under fluid conditions (Thomen et al., 2017; Liu et al., 2019). A study done by Hou et al. (2018) showed that shear flow enhances biofilm formation by increasing the EPS production and strength of the EPS-matrix in *S. aureus*. Their study confirmed the previous speculation on pressure-induced EPS production. It has been suggested that the resulting EPS-matrix serves a protective role in allowing biofilm to recover from mechanical challenges induced by pressure and flow, resulting in more resistant and compressible biofilms (Limoli et al., 2015). Biofilms cultured under shear stress also increased the expression of molecules involved in signal transduction and improved oxygenation, favoring bacterial growth. For instance, Rodesney et al. (2017) found that applied shear stress to biofilm resulted in increased levels of *c-di-GMP* signal in *P. aeruginosa*, which then promote biofilm development. It has been proposed that fluid shear creates a positive feedback loop, initiating biofilm formation by stimulating EPS production and providing nutrients for growth (Zhu et al., 2020).

Another study regarding the dynamic conditions and the influence of species composition using a parallel plate flow chamber showed that the multi-species biofilms, composed of *Streptococcus oralis* and *Actinomyces naeslundii*, became ten times more resistant to compressive forces than single-species biofilms (Paramonova et al., 2009). These findings were confirmed by a study done by Dunsmore et al. (2002). They experimented on the *Desulfovibrio* species and found differences in the viscoelastic properties of biofilms under varying fluid velocities. High-flow biofilm clusters exhibited higher elastic modulus with a greater propensity to return to original shape after the exertion of strain, while low-flow biofilm was less rigid with irreversible damage to the structure after strain (Dunsmore et al., 2002). Overall, biofilm grown in dynamic conditions tends to be more elastic, more resistant, denser in matrix proteins and EPS as reported elsewhere (Lemos et al., 2015; Araújo Paula et al., 2016).

As indicated, the human mouth is a good example to study the influence of dynamic flow conditions on bacterial adhesion and biofilm formation as there is continuous salivary flow. Various artificial mouth models containing a continuous flow of saliva have been applied while mimicking the temperature, pH, and sucrose supply in the oral cavity. In a recent study done by Santos et al. (2019), they compared biofilm formation in static and semi-dynamic conditions by utilizing McBain saliva. They found that the biofilm viability was significantly lower for the static model than the semi-dynamic counterpart (Santos et al., 2019). Many previous studies confirm the findings that dynamic flow can increase the thickness of biofilm growth, (Paramonova et al., 2009), biofilm density (Lewandowski and Beyenal, 2003), biofilm elasticity, resistance, tolerance to antibiotics (Kostenko et al., 2010), and biofilm rigidity (Stoodley et al., 2001).

Despite previous findings on how dynamic conditions favors biofilm growth and viability, many studies reported contradictory findings (Fink et al., 2015; Hizal et al., 2017; Song W.S. et al., 2017; Oder et al., 2018). A study done by Stepanovic et al. (2001) showed *S. epidermidis* and *S. aureus*, tested under dynamic conditions, resulted in a lower degree of biofilm formation. Another study done by Song W.S. et al. (2017) showed that periodontal biofilms composed of *Fusobacterium nucleatum* and *P. gingivalis* formed loose biofilms when cultured under dynamic fluid, thereby exhibiting less bacterial count. It suggested that higher than a certain degree of hydrodynamic conditions may cause recirculating eddies that can disrupt cohesive bonds between biofilms and surfaces (Picioreanu et al., 2000). In contrast, other studies have found no significant differences in biofilm production by oral bacterium *A. naeslundii* (Paramonova et al., 2009) or *Actinomyces oris* (Ding et al., 2010) under shear stress. Differences in those results could be attributed to the type of bacterial species, type of flow (laminar or turbulent), the magnitude of shear stress applied, and surface properties.

**FIGURE 5:**
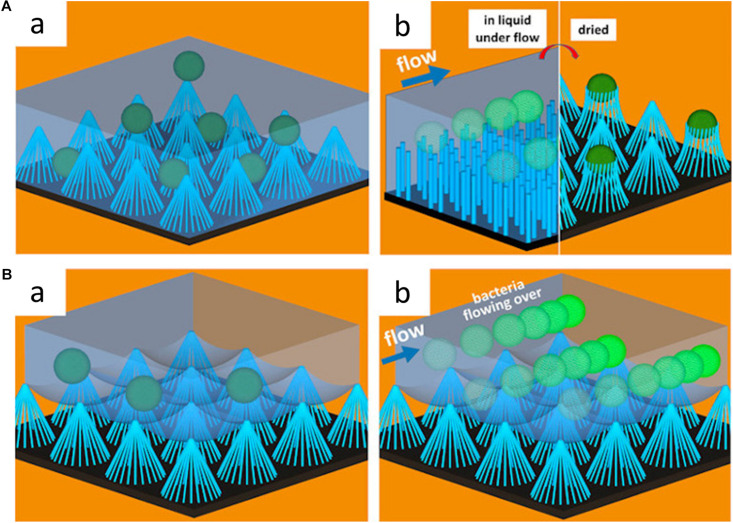
Schematic illustration of the effects of surface wettability on bacterial adhesion under dynamic conditions. **(A)** Schematic illustration of *S. aureus* adhesion on hydrophilic nanopillar surfaces under static and fluid conditions. In the static condition, bacteria adhere to both the nanopillar tips and troughs, while bacteria adhere to only the nanopillar tips under fluid conditions. Adapted from Hizal et al. (2017). **(B)** Schematic illustration of *S. aureus* adhesion on hydrophobic nanopillar surfaces under static and fluid conditions. In the static condition, the bacteria float over the entrapped air layer, and bacteria are swept away by fluid flow. Reprinted with permission from Hizal et al. (2017) Nanoengineered Superhydrophobic Surfaces of Aluminum with Extremely Low Bacterial Adhesivity. ACS Appl Mater Interfaces 9, 12,118–12,129. Copyright© 2017, American Chemical Society.

These hydrodynamic conditions can also significantly affect the anti-adhesion properties of a surface. Under flow conditions, for instance, the anti-adhesion properties of superhydrophobic surfaces can be more pronounced. Hizal et al. (2017) compared the number of colony-forming units adhering to sample surfaces with different surface topography and surface hydrophobicity under both static and flow conditions. They found that hydrophobic nanopillared surfaces exhibited improved reduction of *S. aureus* and *E. coli* adhesion under flow conditions compared to static conditions (Hizal et al., 2017). The high fraction of trapped air in the hydrophobic layer minimized the contact of bacterial suspension to the surface. The air layer entrapped at the interface between bacteria and nanopillar structure helps floating bacteria at the surface to be easily driven off by flow (see Figure 5). The decreased contact areas resulted in the enhancement of the hydrodynamic detachment force and mitigated bacteria attachment (Hizal et al., 2017). Studies regarding fluid dynamics and its effects on bacterial adhesion and biofilm formation are summarized in Table 6.

**TABLE 6:** Influence of fluid dynamics biofilm growth and characteristic.

Microorganism	Flow conditions tested	Influence on biofilm growth and characteristic	References
*Bacillus cereus*	Shear stress	Biofilm density increased with an increase in shear stress, while the biofilm thickness decreased	Lemos et al., 2015
*P. aeruginosa*	Shear stress	Shear acts as a cue for surface adhesion and activates *c-di-GMP* signaling, increasing the strength of bacterial adhesion	Rodesney et al., 2017
*E. coli*	Shear stress	Low shear stress resulted in uniform biofilm growth while high stress resulted in biofilm growth from the edge toward the center of the channel	Thomen et al., 2017
*S. aureus*	Shear stress	Increased shear stress increases EPS production, resulting in higher biofilm density	Hou et al., 2018
*Thalassospira* strain	Shear stress	Flow velocity of 1.66 mm/s is the optimal rate for fast and strong biofilm adhesion, while too high shear stress prevented biofilm formation and removed adhered biofilms	Liu et al., 2019
*Actinobacillus succinogenes*	Shear stress	Enhanced bacterial metabolic activities and biofilm viability under high shear condition	Mokwatlo et al., 2020
*E. coli* and *S. aureus*	Shear stress	Higher shear force was required to reduce bacterial adhesion on the hydrophilic surface compared with the hydrophobic surface	Zhu et al., 2020
*E. coli*	Laminar and turbulent flow	Turbulent flow significantly reduced bacterial adhesion compared with laminar flow	Fink et al., 2015
*P. fluorescens*	Linear flow	High flow velocities resulted in thinner biofilms with higher cell densities and contents of the matrix/extracellular polysaccharides	Araújo Paula et al., 2016
*E. coli*	Laminar and turbulent flow	Laminar flow promotes biofilm growth over a 72 h period, while turbulent flow after 48 h causes reduction in biofilm biomass	Oder et al., 2018
*S. aureus* and *E. coli*	Static and dynamic	Greater reduction in bacterial adhesion to hydrophobic surfaces under flow conditions	Hizal et al., 2017
*F. nucleatum* and *P. gingivalis*	Static and dynamic	Loosely formed biofilm and reduced bacterial count were observed under the dynamic condition	Song W.S. et al., 2017
Biofilm from human saliva	Static and dynamic	Biofilm viability in the static model was lower compared with the semi-dynamic model	Santos et al., 2019

As such, fluid flow dynamics can alter not only the bacterial behaviors on surfaces but also the anti-adhesion activities of surfaces. This important yet often overlooked factor can play an important role in understanding the pathogenesis of bacterially-induced infectious diseases and eradicating them. Further exploration of the influence of hydrodynamics on bacterial colonization on surfaces with various surface properties will contribute to developing novel antifouling strategies. Therefore, the impact of flow dynamics needs to be considered when evaluating the effect of surface properties on bacterial adhesion.

### Bacterial Motility

Microorganisms can be broadly divided into motile and non-motile bacteria. Since these two have clear differences in the structural and behavioral patterns, their adhesion to the substrate surface could be greatly varied. Unlike non-motile bacteria, motile bacteria have a chemotaxis transducer, a protein that makes bacteria sensitively detect physical and chemical changes in the surrounding environment, and a flagellum, a surface appendage that confers bacteria active locomotion. Using these characteristics, motile bacteria can actively search, sense, and accumulate toward a favorable environment or move away from unfavorable environments. Therefore, the binding mechanism of the non-motile bacteria to substrate surfaces may heavily rely on gravitational sedimentation, while the motile bacteria with locomotion may exhibit different adhesive reactions to the same substrate surface.

Since the diffusion coefficient of motile bacteria is more than 3 times higher than paralyzed cells, it contributes more to the adhesion rate, increasing the chance to encounter the substrate surface in the same volume of fluid, thereby increasing the number of adherent cells per unit area when favorable. The flagella also act as an anchor to firmly attach cells to the substrate by electrostatic attraction. Gram-positive bacteria contain long glycan chains in their membrane which make the body negatively charged and hydrophilic, so they hardly move when attached to the hydrophobic glass surface. In contrast, motile bacterial cells (mostly Gram-negative bacteria) rarely adhere and rather move freely on the hydrophilic glass surface, indicating that hydrophobic surfaces may accommodate motile bacteria more efficiently as reported elsewhere (Jindai et al., 2020). In addition, attachment of *E. coli* with or without flagellum was compared on PDMS substrate with an array of hexagonal features (designated as “HEX” pattern). The *filC* mutant (without flagellum) showed a reduced adhesion to the HEX pattern on PDMS (vs. wild type *E. coli* with flagellum), suggesting that the flagellum of *E. coli* played a crucial role in the attachment process (Friedlander et al., 2013).

As discussed earlier, electrostatic interaction and the tangential shear force between the cell and the surface are important factors affecting bacterial response to surfaces. It has been suggested that the effect of ionic strength and flow rates on bacterial adhesion could be varied between motile and non-motile bacteria. McClaine and Ford (2002) showed that the rate of adhesion of *E. coli* motility deficient mutant strain was 4–10-fold lower than that of *E. coli* wild type strain in a low ionic strength medium. They also investigated the effect of flagella rotation direction on the surface adhesion. In *E. coli*, counterclockwise flagellar rotation causes runs, and clockwise flagellar rotation causes tumbles (Manson, 2010). Interestingly, bacteria with counterclockwise rotating flagella showed a similar adhesion behavior to *E. coli* wild type strains, while “Tumble” cells, that rotate only clockwise, adhered at a much lower rate (McClaine and Ford, 2002). This suggests that the rotation of the flagella in the clockwise and counterclockwise directions has an important effect on cell adhesion. Also, de Kerchove and Elimelech (2008) reported that the initial attachment rate of non-motile bacteria decreased with reduced flow rate, rather than ionic strength. In contrast, the binding of motile bacteria increased significantly with increased ionic strength and a reduced flow rate. Such enhanced attachment rate of motile bacteria with increasing ionic strength indicates that electrolytic concentration significantly affects the bacterial motility as the kinetic mechanism of the flagella motor takes place in a complex electrostatic interaction. Therefore, it is necessary to carefully consider the ionic strength and flow rate of the buffer depending on the type of bacteria and its motility when interpreting the effect of surface properties on bacterial adhesion.

As shown in many studies, Gram-negative (mostly motile) bacteria showed higher cell adhesion and growth rates in all stiffness than Gram-positive (non-motile) bacteria. However, some studies showed that bacterial adhesion to surface stiffness tends to be quantitatively similar regardless of bacterial motility. It has been reported that the colony density of non-motile *S. epidermidis* and motile *E. coli* increased linearly as the substrate stiffness increased over the range of 1 MPa < *E* < 100 MPa (Lichter et al., 2008). Bakker et al. (2003) also reported that marine bacteria adhere more to polyurethane (PU) substrates with higher stiffness under certain flow chamber conditions. In addition, Saha et al. (2013) demonstrated that both non-motile *L. lactis* and motile *E. coli* had a surface coverage of about 17–31% higher on a harder cross-linking film (150 kPa) compared to a softer non-crossing (30 kPa). However, non-motile *L. lactis* was found to grow slowly on both films regardless of stiffness, while motile *E. coli* showed faster growth on soft films. Similarly, Kolewe et al. (2015) showed that the number of adhered cells increased when the stiffness of the hydrogel (PEGDMA and Agar) increased in both motile *E. coli* and non-motile *S. aureus* regardless of incubation time in the range of 44.05 kPa < *E* < 6489 kPa. In another study, Song F. et al. (2017) compared the adhesion of motile *E. coli* and *P. aeruginosa* on soft and hard PDMS surfaces. They reported that *E. coli* exhibited higher motility on the surface of hard PDMS (2.6 MPa) than soft PDMS (0.1 MPa), and the flagellar motor protein motB played an important role in response to PDMS stiffness during initial adhesion (Song F. et al., 2017).

As above, the behavior of motile bacteria contrasts with non-motile bacteria. Bacterial surface appendages such as flagella can play an important role in the adhesion by inducing a more dynamic response of motile bacteria to surface properties than non-motile bacteria. Once bacteria adhere to surfaces, motile bacteria can settle biofilms faster than non-motile bacteria by attracting free bacteria through chemotaxis and quorum sensing (Gutman et al., 2013). Most of the natural and manmade systems are an open ecosystem composed of various microorganisms with different sizes, shapes, cell types, and motility. Even on the same surface, it is possible to induce a contradictory reaction to the initial deposition rate depending on the cell’s motility. Therefore, it is necessary to carefully design the characteristics of the substrate according to the characteristics of the target majority of bacteria.

## Implication for Developing New Anti-Fouling Strategies

The antibacterial surfaces can be created by impregnating bactericidal elements in their structure or coating the surface with a durable antimicrobial material. Although these antibacterial surfaces can prevent the formation of biofilm mainly by killing surrounding bacteria, the continuous release of the antibacterial elements to the surrounding environment may lead to bacterial resistance to these specific elements. Also, it may affect the long-term efficiency of these surfaces (Nino-Martinez et al., 2019). To overcome this issue, alternative approaches have been proceeded; developing a surface that (i) can hinder bacterial adhesion at its early stages that eventually alter biofilm formation (Zhang et al., 2018; Chi et al., 2019), (ii) physically disrupt membrane of adhered bacteria (Reed et al., 2019; Wandiyanto et al., 2019), or (iii) exhibits multifunctional antiadhesion/antibacterial properties (Balaure et al., 2016; Borcherding et al., 2019).

Laser-based techniques used to modify surface topography can be a powerful technique for controlling bacterial colonization. As an alternative approach to antibacterial coating, Laser-induced periodic surface structures (LIPSS) was introduced to inhibit bacterial attachment by utilizing ultrashort laser pulses to create nanostructures on most surface materials. One study done using laser structured steel samples were subjected to microbial adhesion tests in a dynamic flow chamber with *E. coli* and *S. aureus*. The result showed an anti-adhesion effect for *E. coli* but not for *S. aureus*. Differences in adhesion were attributed to the geometry and colonization characteristics of *S. aureus* (Epperlein et al., 2017). Another study also used femtosecond laser irradiation to produce nano-ripples. They exhibited reduced adhesion of *E. coli* on nano-ripples and micro-grooves. The deep grooves of the nanostructure were conducive to the rupture of bacteria cells (Luo et al., 2020). To confirm these studies, Siddiquie et al. (2020) used both LIPSS and multiscale structures (MS) to create microtopographies on PDMS and PU elastomers to investigate bacterial retention. They found both LIPSS and MS topographs reduced *E. coli* adhesion by >89% (Siddiquie et al., 2020). Many recent studies have also confirmed the bactericidal actions of laser-induced topography (Kudryashov et al., 2018; Jalil et al., 2020; Ma et al., 2020).

The combination of mechano- and chemo- bactericidal properties can be a powerful and effective tool to develop hybrid anti-biofilm solutions. Coating of Si nano-ripples with Se, TeO_2_, Sb_2_O_3_, and Ag NPs was able to damage the bacterial DNA by producing reactive oxygen species. The combined chemical toxicity of nanoparticles and mechanical damage by nanoscale structures synergistically increased the antibacterial properties of the surface and effectively reduced *S. aureus* biofilms formation (Saraeva et al., 2020). Utilizing the knowledge of antibacterial nanoparticles and topography, Qian et al. (2017) prepared a multilayer antibacterial film with hierarchical nanostructures and superhydrophobicity using polydopamine (PDA) and silver nanoparticles. This Ag-deposited surface (SS) film exhibited bifunctional antiadhesion and antibacterial activities against *E. coli* and *S. aureus*, which may allow for lower cytotoxicity compared to the surface with only antibacterial components (e.g., silver nanoparticles). They also quantified the biofilm thickness and coverage area; the addition of Ag nanoparticles alone failed to control biofilm thickness after 3 days while the SS successfully prevented the formation of biofilm. The results of this study highlight the synergistic effect of Ag nanoparticles and superhydrophobic surfaces (Qian et al., 2017). Similarly, another study paired bactericidal copper ions with superhydrophobic surfaces. The hybrid approach exhibited both anti-adhesive and bactericidal properties, suggesting short and long-term antibacterial protection against the *Synechococcus* species. The superhydrophobic and Cu-enriched surfaces exhibited lower bacterial adhesion and higher antibacterial properties than either parameter alone (Ellinas et al., 2017). Using inorganic nanoparticles in combination with antibiotics, a 10,000-fold reduction of bacterial cells was achieved against *S. aureus* and *P. aeruginosa* biofilms. This was created using matrix-assisted pulsed laser evaporation to create a thin film consisting of a magnetite/salicylic acid/silica shell with an antibiotic coating (Mihaiescu et al., 2013).

While nanostructured surface-based approaches could open new perspectives for developing antibiofilm surface materials, nanoscience-based surface modifications are still at an early stage of research. Several nanoparticle surface coating solutions have been uncovered but very few hybrid approaches have been developed. Thus, more investigation is needed for these to become promising candidates for the development of novel materials for biofilm inhibition and the exploitation of nanoscience-based technologies.

## Concluding Remarks

Bacterial adhesion and biofilm formation is a complex process controlled by the interplay between physicochemical, mechanical, topographical surface properties, bacterial characteristics, and environmental conditions. This review provides a comprehensive briefing and insight into the characterization of parameters that affect bacterial adhesion. Much of the research done to date did not consider the implications of multiple surface parameters, bacterial motility, or surrounding hydrodynamic conditions to the bacterial sensing and binding behavior on surfaces. Importantly, in reality, bare surfaces are covered by conditioning films of organic and inorganic matters before bacteria bind, thereby also affecting bacterial binding behaviors significantly. Therefore, rather than focusing on a single surface parameter and its effect on adhesion, scientific efforts assessing the impact of multiple surface parameters on bacterial adhesion are essential to further advance knowledge. Furthermore, the temperature may play an important role as an environmental factor governing bacterial adhesion; it induces not only the complex biological functions (e.g., gene expression, piliation, cell wall hydrophobicity) but also alters the properties of the substrate surface (e.g., wettability, surface charge density). Therefore, more research reflecting the corresponding environmental conditions, bacterial types, and surface properties are needed to develop efficient and reliable antibiofilm solutions. Nanopatterning of surfaces and their hybrid approach with bactericidal compounds have great prospects to provide more advanced solutions for biofilm-related fouling in medical or industrial settings.

## Author Contributions

GH planned and constructed the study. SZ, MB, AD, H-EK, LH, and JH contributed to data acquisition and interpretation. SZ, MB, AD, H-EK, LH, JH, and GH drafted the manuscript and critically revised the manuscript. All authors gave their final approval and agreed to be accountable for all aspects of the work.

## Conflict of Interest

The authors declare that the research was conducted in the absence of any commercial or financial relationships that could be construed as a potential conflict of interest.
